# Intravital Whole‐Process Monitoring Thermo‐Chemotherapy Via 2D Silicon Nanoplatform: A Macro Guidance and Long‐Term Microscopic Precise Imaging Strategy

**DOI:** 10.1002/advs.202101242

**Published:** 2021-06-24

**Authors:** Doudou Huang, Guangxing Wang, Jingsong Mao, Chunlei Liu, Zhongxiong Fan, Yunrui Zhang, Bei Zhang, Yang Zhao, Cuixia Dai, Yaqin He, Heng Ma, Gang Liu, Xiaoyuan Chen, Qingliang Zhao

**Affiliations:** ^1^ State Key Laboratory of Molecular Vaccinology and Molecular Diagnostics Center for Molecular Imaging and Translational Medicine School of Public Health Xiamen University Xiamen 361102 China; ^2^ Department of Radiology Xiang'an Hospital of Xiamen University Xiamen 361102 China; ^3^ Laboratory of Translational Medicine Medical Innovation Research Division of Chinese PLA General Hospital Beijing 100853 China; ^4^ Department of Biomaterials College of Materials Research Center of Biomedical Engineering of Xiamen & Key Laboratory of Biomedical Engineering of Fujian Province & Fujian Provincial Key Laboratory for Soft Functional Xiamen University Xiamen 361102 China; ^5^ Department of Mechanical and Electrical Engineering Xiamen University Xiamen 361102 China; ^6^ College of Physics Shanghai Institute of Technology Shanghai 201418 China; ^7^ Department of Colorectal Surgery General Hospital of Ningxia Medical University Yinchuan 750004 China; ^8^ Department of Physiology and Pathophysiology School of Basic Medical Sciences Fourth Military Medical University Xi'an 710032 China; ^9^ Yong Loo Lin School of Medicine and Faculty of Engineering National University of Singapore Singapore 117597 Singapore; ^10^ State Key Laboratory of Molecular Vaccinology and Molecular Diagnostics Center for Molecular Imaging and Translational Medicine School of Public Health Xiamen University Xiamen 361102 China; ^11^ Shenzhen Research Institute of Xiamen University Shenzhen 518063 China

**Keywords:** 2D silicon nanosheets, microvascular quantification, multimodal optical imaging, optical coherence tomography angiography, thermo‐chemotherapy

## Abstract

Tumor angiogenesis is a complex process that is unamenable to intravital whole‐process monitoring, especially on microscopic assessment of tumor microvessel and quantifying microvascular hemodynamics before and after the nanotherapeutics, which hinder the understanding of nanotheranostics outcomes in tumor treatment. Herein, a new photoacoustic (PA) imaging‐optical coherence tomography angiography (OCTA)‐laser speckle (LS) multimodal imaging strategy is first proposed, which is not only able to precisely macro guide the thermo‐chemotherapy of tumor by monitoring blood oxygen saturation (SaO_2_) and hemoglobin content (HbT), but also capable of long‐term microscopic investigating the microvessel morphology (microvascular density) and hemodynamics changes (relative blood flow) before and after the nanotherapeutics in vivo. Moreover, to realize the tumor thermo‐chemotherapy treatment based on this novel multimodal imaging strategy, a 2D 5‐fluorouracil silicon nanosheets (5‐Fu‐Si NSs) therapeutic agent is designed. Furthermore, 2D high‐resolution tumor microvascular images in different stage display that tendency of the thermo‐chemotherapy effect is closely associated with tumor angiogenesis. Taken together, the investigations establish the fundamental base in theory and technology for further tailoring the novel specific diagnosis and treatment strategy in tumor. More importantly, this technique will be beneficial to evaluate the tumor microvascular response to nanotherapeutics at microscale.

## Graphical abstract

A promising biocompatible nanotheranostics agent based on chemotherapeutic drug 5‐fluorouracil and 2D silicon nanosheets is developed for synergistic thermo‐chemotherapy strategy. The tumor tissue functional parameters, including SaO_2_, HbT, micro microvascular morphology, and hemodynamics, are dynamically monitored by multimodal PA/ OCTA/ LS imaging strategy to accurately evaluate overall therapeutic effects. This novel strategy exhibited prominent therapeutic efficiency on malignant glioma *U87‐MG*.

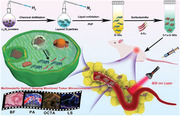

## 1 Introduction

Nanotheranostics method has been widely utilized in the diagnosis and treatment of a great deal of diseases.^[^ ^1^, ^2^ ^]^ One of the most hotly researched spots of nanotheranostics is tumor diagnosis and treatment.^[^ ^3^ ^]^ Recently, to prolong the bloodstream circulation time of nanotheranostics, enhance their tumor enrichment, and improve the therapeutic efficacy of cancer, nanotheranostics of surface functionalization with surfactants and stabilizers have been widely developed.^[^ ^4^, ^5^, ^6^ ^]^ However, the size and morphology of such functional nanotheranostics possess a larger change than those of unfunctionalized nanotheranostics, thus receding in the uptake of nanotheranostics into the deep biological targets within the sophisticated structure of cells or tissues. Notably, the hydroxyl group surface‐functionalized quantum dots (QDs) could significantly reduce their nonspecific binding to plasma opsonin, and inhibit phagocytosis by the reticuloendothelial system (RES) compared with PEGylated QDs, which could prolong the blood circulation time.^[^ ^7^ ^]^ Therefore, it is imperative to develop a dual‐functional surfactant that could prolong the circulation lifetime and maintain the integrity of structures in vivo. We focus on two specific objectives. One is enhancing the nanotheranostics agents (NTAs) circulation time for better photothermal therapy (PTT), which is a well‐known treatment modality involving PTT agents, and efficiently convert near‐infrared (NIR) light energy into ablative heat to kill cancer.^[^ ^8^, ^9^, ^10^ ^]^ The other is to improve chemotherapeutic effect. Previously, It has been reported that the hydroxyl group surface‐functionalized QDs could significantly reduce their nonspecific binding to plasma opsonin, and inhibit phagocytosis by the RES compared with PEGylated QDs, which could prolong the blood circulation time.^[^ ^7^ ^]^ Enlightened by the above point, we speculated that whether it is possible to integrate the surfactant‐like ability with a chemotherapeutic drug to endow the theranostic nanodrug with outstanding biological stability and prolonged circulation time. Although PTT strategy is direct and rapid, the effect of this treatment strategy is weakened in the deep tumor due to the laser penetration depth limitation. Inversely, chemotherapy can occur in tumor through the blood circulation in vivo. Aiming at this goal, a surfactant‐like chemotherapeutic drug 5‐fluorouracil (5‐Fu) with a hydrophilic moiety (one fluorine on pyrimidine) and a hydrophobic moiety (pyrimidine rings) was selected to introduce on the initial Si NSs by electrostatic interactions. This strategy could not only improve the biological stability and physiochemical stability of 2D silicon nanosheets (Si NSs) but also prolong the circulation lifetime. In addition, 5‐Fu is a promising pyrimidine analog and an inhibitor of thymidylate synthase with low toxicity. It has been widely used to treat many solid tumors by inhibiting the synthesis of DNA and RNA in cancer cells.^[^ ^11^ ^]^ Therefore, 5‐Fu could play a dual‐drug synergy inhibition of DNA and RNA synthesis to improve chemotherapeutic effect. In addition, previous reports have confirmed that single‐layer 2D materials possess the advantages of high specific surface area, stability, better thermal conductivity, and high light transmittance.^[^ ^12^, ^13^, ^14^ ^]^ Moreover, 2D nanomaterials show great potential and prospects in nano‐medicine, energy storage, electromagnetic materials, and optical materials.^[^ ^15^, ^16^, ^17^ ^]^ Silicon is widely distributed in many organs (such as bones, hair, and skin). Under physiological conditions, the silicon is gradually transformed into non‐toxic orthosilicic acid [Si(OH)_4_]. On this basis, it is widely used in various biomedicines such as drug delivery, biosensors, and imaging,^[^ ^18^, ^19^ ^]^ and is generally considered to be biocompatible and biodegradable in biomedical applications. In particular, silicon nanostructures such as silicon nanowires or porous silicon have shown interesting applications in many aspects due to their safety and semiconductor properties.^[^ ^20^, ^21^ ^]^ Hence, it is particularly meaningful to combine 2D Si NTAs PTT and chemotherapy drug strategies to treat tumor.

In order to determine the optimal treatment strategy, the tumor growth situation before and after treatments should be effectively and accurately evaluated. Specifically, tumor angiogenesis is necessary for the continued survival and development of tumor cells, and plays an important role in their growth, maintenance, invasion, and metastasis.^[^ ^22^, ^23^ ^]^ The evaluation of tumor vascularization is also a potential indicator to estimate and quantify the PTT efficacy of cancer.^[^ ^24^ ^]^ Therefore, combining the macro imaging guidance with microscopic precise imaging monitor strategy to non‐invasively feed back the tumor microvascular development, evaluate overall therapeutic effects, and quantify the functional parameters before and after treatment has vital practical significance for tumor therapy. Several frequently used molecular imaging approaches such as micro‐magnetic resonance imaging (μMR),^[^ ^25^ ^]^ micro‐computed tomography (μCT), and fluorescence microscope imaging are utilized to study angiogenesis of tumors in both clinical and preclinical models.^[^ ^26^, ^27^ ^]^ However, μMR and μCT cannot show a single vessel due to their limited resolution.^[^ ^28^ ^]^ Fluorescence microscopy requires entire labeling of the vasculature through exogenous contrast agents, which leads to limitations in successive imaging studies in vivo.^[^ ^29^, ^30^ ^]^ Besides, the routine studies on the nanotherapeutics of tumor mainly focus on one or several specific points of the tumor itself, such as tumor size, tumor volume, and various image signal intensity, but lack of combining macro with micro systematic structure and functional information research, such as hemodynamic study of tumor. In this regard, we introduced a burgeoning hybrid imaging modality, dual‐wavelength 3D PA imaging, to provide SaO_2_ and HbT of the tumor during the growth process, which could be used as early vital indicators for macro monitoring tumor development and guiding treatment.^[^ ^31^, ^32^, ^33^, ^34^ ^]^ In addition, the change in microvascular hemodynamics was precisely monitored during tumor early growth and NTAs treatment using the emerging OCTA technology. Meanwhile, we introduced LS to detailedly estimate and quantify the dynamic response of tumor vasculature as well as the treatment efficacy of cancer in real‐time. Furthermore, the understanding of the influence of vascular morphometrics changes caused by these NTAs in tumor development is limited. As a result, combining the PA macro guidance with microscopic OCTA and LS precise monitoring conception can be used as a new auxiliary guidance strategy for the current nanotherapeutic.

Following the conception above, in this work, we reported a simple, rapid, and scalable approach coupling chemical delithiation and cryogenic exfoliation to exploit tiny 2D Si NSs with the trade‐off between stability and degradability, which will satisfy the requirements of both storage and biodegradability for practical tumor treatment. Besides, benefiting from the small size and high photothermic performance, the developed Si NSs were loaded with a broad‐spectrum anticancer drug 5‐Fu to gain high stability 5‐Fu‐Si NSs. Subsequently, a nude mice glioma tumor model bearing *U87‐MG* cells was established using a dorsal skinfold window chamber, and the changes in SaO_2_ and HbT were quantified by PA imaging. Furthermore, we exploited high‐resolution OCTA and LS to achieve long‐term monitoring of the tumor microvascular during the tumor growth and thermo‐chemotherapy. More importantly, based on the OCTA and LS images, we calculated the tumor microvascular density (MVD) and relative blood flow (RBF) during pre‐therapy and post‐treatment, respectively.

**Scheme 1:**
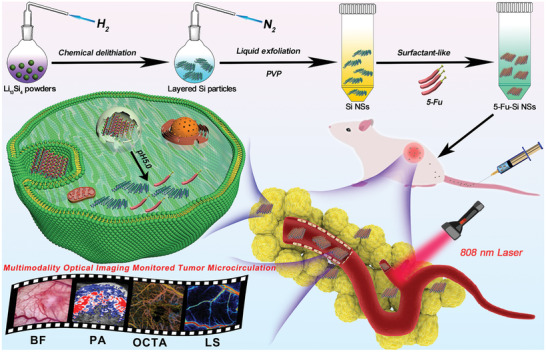
Schematic representation of synergistic cancer thermo‐chemotherapy implemented by 2D silicon nanosheets loaded with anticancer drug 5‐Fu for PA imaging‐guided phototherapy and OCTA/LS long‐term imaging‐monitored tumor microvascular strategy

To the best of our knowledge, we firstly reported that in vivo long‐term dynamically estimate microvasculature morphometric changes and quantify microvascular hemodynamics during tumor growth and thermo‐chemotherapy with 2D NTAs in the *U87‐MG* glioma mice model, combining macro guidance and microscopic precise monitoring multi‐optical PA/OCTA/LS imaging strategy (**Scheme** **1**). This feasibility study would be significant to current nanotherapeutics in cancer prevention, diagnosis, and treatment.

## 2 Results and Discussion

### 2.1 Synthesis and Characterization of 5‐Fluorouracil Silicon Nanosheets (5‐Fu‐Si NSs)

**Figure 1:**
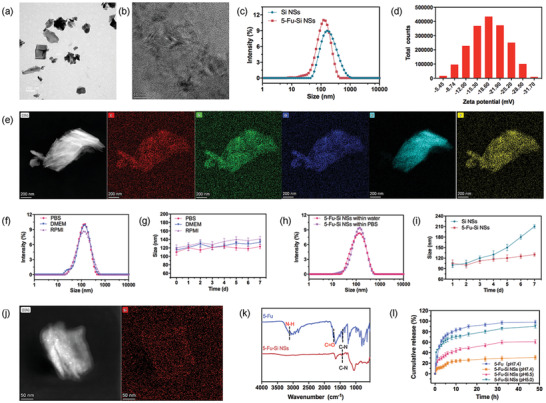
Synthesis and characterization of the 5‐Fu‐Si NSs. a) TEM image of 5‐Fu‐Si NSs. b) TEM image of the Si NSs lattice. c) Diameter distribution of Si NSs and 5‐Fu‐Si NSs. d) Zeta potential of 5‐Fu‐Si NSs. e) TEM‐EDS mapping images of 5‐Fu‐Si NSs dispersed in water. f) Diameter distribution of the 5‐Fu‐Si NPs dispersed in PBS DMEM medium with 10% FBS and RPMI for 24 h. g) Diameter distribution of the 5‐Fu‐Si NPs dispersed in PBS DMEM medium with 10% FBS and RPMI for 7 days. h) Diameter distribution of the 5‐Fu‐Si NSs dispersed within water and PBS. i) Diameter distribution of the Si and 5‐Fu‐Si NSs dispersed in PBS for 7 days. j) TEM‐EDS mapping images of Si NSs dispersed in water. k) FT‐IR spectra of 5‐Fu‐Si NSs. l) Cumulative release of 5‐Fu at pH 7.4 and 5‐Fu‐Si NSs at pH 7.4, 6.5, and 5.0 at 37 °C.

In this work, first, 2D Si NSs were synthesized via a simple approach coupling chemical delithiation following the previously reported method.^[^ ^35^ ^]^ And then, the layered Si NSs were liquid exfoliation by PVP‐capped. Subsequently, the 2D 5‐Fu‐Si NSs were synthesized by a classical physical electrostatic forces adsorption mechanism following the previous studies.^[^ ^36^, ^37^ ^]^ In brief, the chemotherapy drug of 5‐Fu could be adsorbed at the surface of 2D Si NSs in order to increase their biocompatibility, stability, and prolong the circulation time in the body. Thereby, the combinational therapy efficiency was improved. To further confirm the formation of 5‐Fu‐Si NSs, transmission electron microscopy (TEM) and dynamic light scattering (DLS) were implemented to measure and observe the morphology and diameter of the 5‐Fu‐Si NSs as illustrated in **Figure** **1**a,c. As displayed in Figure 1c, the mean diameter of monodisperse 2D‐shape Si NSs in DI water was around 120 nm. Besides, the clear lattice of Si NSs was observed by a high‐resolution TEM (FEI, Talos 200s) in Figure 1b. Representative TEM images showed that the 5‐Fu‐Si NSs were monodisperse and the uniform size was 128.9 ± 3.5 nm (Figure 1a), with a relatively narrow distribution (Figure 1c). Benefit from the 5‐Fu modification, Si NSs were more evenly distributed and the DLS size is more suitable for biomedical applications. The elemental mapping of 5‐Fu‐Si NSs demonstrated that the C, N, O, Si, and F elements were successfully enclosed inside 5‐Fu‐Si NSs (Figure 1e). Besides, the FT‐IR spectra confirmed the formation of coordination bonds (Figure 1k). As shown in Figure 1k, 5‐Fu‐Si NSs exhibited an N─H characteristic peak at 3121.30 cm^−1^ and C═O (carbonyl) peak at 1719 cm^−1^, indicating the presence of electrostatic interaction between the 5‐Fu molecules and Si NSs. The surface charge of Si NSs is negative.^[^ ^35^ ^]^ And the surface charge of 5‐Fu is positive. This is further evidence of the presence of electrostatic interaction between the 5‐Fu molecules and Si NSs. Meanwhile, the Zeta potential of 5‐Fu‐Si NSs confirmed by DLS was concentrated at −18.60 Mv (Figure 1d), which makes the whole system a relatively stable nanometer system and can avoid the adsorption and phagocytosis of the RES. Besides, high‐resolution TEM‐energy dispersive spectroscopy (TEM‐EDS) mapping displayed a homogeneous distribution of Si elements in Si NSs (Figure 1j). The content of element Fu accounted for 2.90% in 5‐Fu‐Si NSs. (Figure S3a, Supporting Information). Based on the above results, we successfully synthesized 5‐Fu‐Si NSs with sheet structure.

### 2.2 In Vitro Drug Loading and Releasing Behaviors

In addition, Si NSs have a 2D sheet structure with a high specific surface area. The physical characteristics of Si NSs make them exhibit strong load capacity. This would be very helpful for the packaging and transportation of the drugs. Therefore, Fu was selected as the drug model to evaluate the load capacity of Si NSs. By mixing Fu with Si NSs, the Fu was adsorbed to the Si surface by a weak interaction force, and the calculated loading capacity (LC) was 30.03% according to Equation (1). The excellent drug delivery capacity will facilitate the release of more drugs in the tumor area and play a crucial part in further chemotherapy. Considering that the properties of tumor tissue possess low acidity and higher redox potential, the drug delivery system must remain effective in the tumor environment.

To verify the pH response behaviors of 5‐Fu‐Si NSs, the different pH values of PBS media were utilized to simulate healthy human physiological conditions and tumor cell environmental situations. Next, the amount of Fu released from 5‐Fu‐Si NSs was calculated at different pH conditions. As shown in Figure 1l, the 5‐Fu‐Si NSs showed significant pH‐dependent release behavior. The releasing amount of Fu was gradually increasing with pH value decreased. Intriguingly, at 12 h the 5‐Fu‐Si NSs showed significant pH‐dependent release behavior. When the pH value approached the microenvironment of the tumor (acidic pH 6.5 and 5.0), the accumulated release of Fu was ≈47.33% and ≈71.43%, respectively. Compared with neutral pH (pH 7.4) condition (which simulated normal physiological environment), the accumulated release of it was only ≈25.32% (Figure 1l). This phenomenon could be attributed to the disappearance of the electrostatic interaction between the Si NSs and 5‐Fu molecules with pH value decreased from 7.4 to 5.4.^[^ ^38^, ^39^ ^]^ Hence, our strategy could be expected to delay the drug release under healthy physiological and keep the complete layered nanostructure before being ingested by the tumor cells.

### 2.3 In Vitro Physical Stability

It is important to evaluate the physical stability of 5‐Fu‐Si NSs for further biomedical applications in vivo. As shown in Figure 1f,g, the mean diameter of 5‐Fu‐Si NSs was kept in various aqueous media for 24 h and 7 days without showing significant changes, respectively. In addition, the mean size of 5‐Fu‐Si NSs stored in water and PBS showed no significant change over the 24 h period (Figure 1h). Impressively, the 5‐Fu‐Si exhibited remarkable stability without any phase separation or drug leakage in PBS, DMEM, and RPMI, ensuring their transferring to the tumor area as intact nanostructures in vivo long‐time circulation (Figure 1f–h). However, the Si NSs system without any modifications showed adsorption and deposition, resulting in dimensional instability (Figure 1i). The above results suggested that the 5‐Fu‐Si NSs nano‐system possesses excellent stability and has great potential for further long‐term biomedical applications.

### 2.4 Cytotoxicity and Thermo‐Chemotherapy Assay of 5‐Fu‐Si NSs

**Figure 2:**
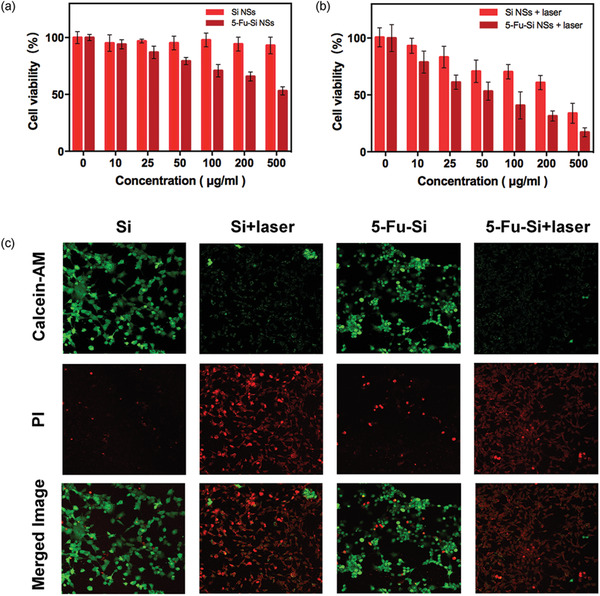
a) The concentration‐dependent cell viability of *U87‐MG* cells treated with Si NSs and 5‐Fu‐Si NSs after 12 h incubation. b) The concentration‐dependent cell viability of *U87‐MG* cells treated with Si NSs plus laser and 5‐Fu‐Si NSs plus laser after 12 h incubation. c) Calcein‐AM (green, live cells) and PI (red, dead cells) staining fluorescent images of *U87‐MG* cells cultured for live‐dead assay to evaluate thermo‐chemotherapy of 5‐Fu‐Si NSs with different treatments.

Next, the low toxicity of nanomaterials is a key factor in their clinical translation before further biomedical applications are investigated. The cytotoxicity of Si NSs was first assessed by MTT assay. The viability of *U87‐MG* cells remained above 94.27 ± 6.1%, showing good biocompatibility, even when the concentration of Si NSs reached 200 µg mL^−1^ (**Figure** **2**a). However, after 24 h incubation with the same concentration of 5‐Fu‐Si NSs, the cell viability fell to 67.45 ± 3.3%. These results showed the efficient tumor cell‐killing ability in chemotherapy, which was concentration‐dependent (Figure 2a). 5‐Fu is a clinical anticancer drug with good killing effect on cancer cells. Here we demonstrated the cell‐killing effect of the corresponding 5‐Fu concentration in Figure S2, Supporting Information. The results showed good tumor cytotoxicity, due to the cell entry velocity that small‐molecule drugs can enter cells more easily and kill tumor cells more quickly than 5‐Fu‐Si NSs. Interestingly, the cell viability of the combination laser groups (Si NSs plus laser and 5‐Fu‐Si NSs plus laser groups) was sharply decreased compared to those without laser at corresponding concentration, indicating that the synergy of thermo‐chemotherapy was much more cell‐killing effective than single chemotherapy (Figure 2b). The killing efficacy of Si NSs plus laser and 5‐Fu‐Si NSs plus laser groups drastically decreased to 60.86 ± 6.20% and 31.48 ± 4.8%, respectively, after 5 min irradiation with 808 nm laser (1.0 W cm^−2^) (Figure 2b). To further investigations, Calcein‐AM (green fluorescence) and PI (red fluorescence) were used to stain living and dead cells, respectively. After incubation with 5‐Fu‐Si NSs for 24 h and laser irradiation (808 nm, 10 min), as shown in Figure 2c, the control group (Si NSs) showed significant green fluorescence, indicating low toxicity of Si NSs. In contrast, cells in the combined PTT groups (Si NSs plus laser and 5‐Fu‐Si NSs plus laser) showed obvious red fluorescence, indicating that thermo‐chemotherapy combined treatment caused extensive cancer cell death and had a superior cell‐killing effect.

### 2.5 In Vitro Evaluation of Photoacoustic and Photothermal Effects

**Figure 3:**
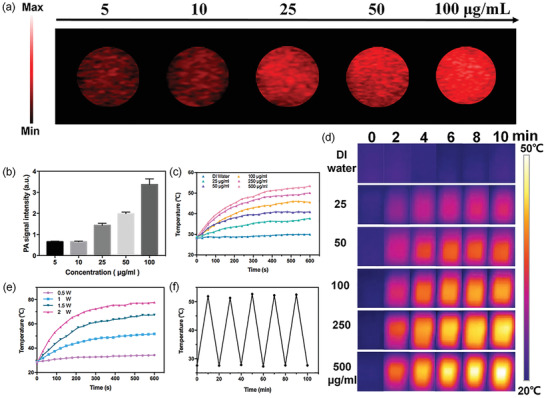
a) PA signal at different concentrations of 5‐Fu‐Si NSs at 808 nm in vitro. b) Corresponding PA signal intensity in (a). c) Photothermal effect of 5‐Fu‐Si NSs at different concentration (25, 50, 100, 250, 500 µg/mL) under 808 nm laser irradiation (1.0 W cm^−2^, 10 min). d) Thermal images of 5‐Fu‐Si NSs at different concentration (25, 50, 100, 250, 500 µg mL^−1^) under 808 nm laser irradiation (1.0 W cm^−2^, 10 min). e) Photothermal effect of 5‐Fu‐Si at 250 µg mL^−1^ in different laser energy. f) Cycling photostability of 5‐Fu‐Si NSs.

Based on the light absorption (Figure S3b, Supporting Information) and low toxicity of 5‐Fu‐Si NSs as displayed above, we further evaluated the potential of 5‐Fu‐Si NSs as contrast agents to enhance PA imaging and simultaneously as photosensitive PTT agents to kill the tumor. As shown in **Figure** **3**a,b, there was a positive correlation between PA signal intensity and 5‐Fu‐Si NSs concentration, showing a strong concentration dependence. In order to further evaluate the tumor photothermal effect of 5‐Fu‐Si NSs in vivo, compared with the DI water (control group), the temperature of 250 µg mL^−1^ of 5‐Fu‐Si NSs group rapidly increased from 28.01 to 46.56 °C in 5 min and reached the maximum temperature of 50.10 °C after 10 min irradiation (Figure 3c). The photothermal images of different concentrations of 5‐Fu‐Si NSs (25, 50, 100, 250, and 500 µg mL^−1^) under the 808 nm laser irradiation (1.0 W cm^−2^) were investigated, respectively (Figure 3d). In addition, we also investigated the release of 5‐Fu from 5‐Fu‐Si NSs (18.75 µg mL^−1^) after laser irradiation (1.0 W cm^−2^) at the wavelength of 808 nm. With the laser irradiation time increase, some 5‐Fu were released from 5‐Fu‐Si NSs (Figure S4, Supporting Information) Next, the temperature rises effect of 5‐Fu‐Si NSs was also investigated under the irradiation of different laser power with the concentration of 250 µg mL^−1^ (Figure 3e). Those findings showed excellent PTT ability of 5‐Fu‐Si NSs. And its temperature rises effect was positively associated with the laser power. Subsequently, the photothermal stability of the 5‐Fu‐Si NSs was also investigated. As shown in Figure 3f, after five heating/cooling cycles, there was even no effect changed on the photothermal conversion behavior. These results exhibited that 5‐Fu‐Si NSs have outstanding photothermal stability before and after laser irradiation. Furthermore, the calculated photothermal conversion efficiency can reach 45.8%, which was much higher than those previously reported.^[^ ^40^, ^41^ ^]^ These results suggested that 5‐Fu‐Si NSs can be used as a potential PTT agent.

### 2.6 In Vivo Evaluation of PA Imaging and Photothermal Effects

**Figure 4:**
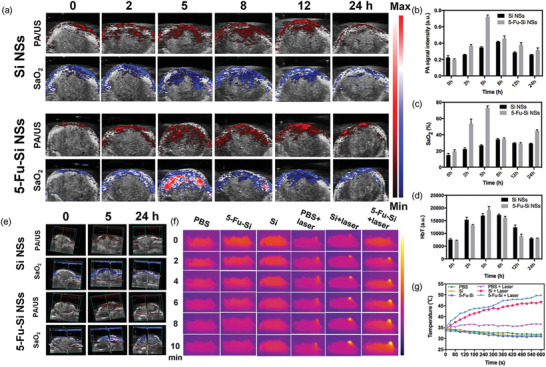
a) In vivo PA imaging in the tumors at different time points after i.v. injection of Si NSs and 5‐Fu‐Si NSs. b) PA intensity of tumors at different time points. c) SaO_2_ intensity of tumors at different time points. d) HbT signal intensity of tumors at different time points. e) In vivo 3D reconstruction PA imaging in the tumors at different time points of Si NSs and 5‐Fu‐Si NSs. f) Thermal images of *U87‐MG* tumor‐bearing mice in different groups under 808 nm laser irradiation (1.5 W cm^−2^). g) Quantitative analysis of temperature changes in tumor area at different time‐points.

In vitro PA contrast agent and PTT agent verification prompted, we continued to carry out in vivo experimental studies. As shown in **Figure** **4**a (top row) and Figure 4b, the PA signal was acquired at 0 h before administration. And then, the PA signal in the tumor area was significantly gradually increased after i.v. injection of Si NSs (50 mg kg^−1^) into the *U87‐MG* tumor model at 2, 5, 8, 12, and 24 h, respectively. The same phenomenon was also observed from the 5‐Fu‐Si NSs (50 mg kg^−1^) at the three‐row in Figure 4a. As shown in Figure 4b, the PA signal intensities in tumor regions gradually increased with the time prolonging. The PA signals of 5‐Fu‐Si NSs and Si NSs reached their peak after injection of 5 and 8 h, respectively. Notably, as depicted in Figure 4b, after injection of 5 h, the PA signal intensity of 5‐Fu‐Si NSs was higher than that of Si NSs. And, the peak PA signal of 5‐Fu‐Si NSs was 3.6 times larger than the initial PA signal before the injection of 5‐Fu‐Si NSs into the corresponding tumor area (Figure 4b). In addition, the peak concentration of SaO_2_ at 5 h was 3.76 times higher than the initial concentration of SaO_2_ before the injection of 5‐Fu‐Si NSs (Figure 4c). The HbT in different time points was also evaluated, which indicated that it had the ability to improve the deoxygenation environment (Figure 4d). These results fully demonstrated that Fu modified Si NSs reached the tumor site much faster due to the uniform dispersion and stability, and also showed that the EPR effect enhanced permeability and retention. 5‐Fu‐Si NSs can significantly enhance PA signal intensity in tumor sections, which is due to the abundant blood vessels, wide vascular wall space, poor structural integrity, and absent lymphatic reflux. The enhancement of PA signals in the tumor region was still observed after injection of 24 h, suggesting that the relatively long cycle time of 5‐Fu‐Si NSs in vivo may be related to the surface modification of Fu molecules. The 3D distribution of PA signal intensity and SaO_2_ in tumor area at different time points (0, 5, and 24 h) were also provided after injection of Si NSs and 5‐Fu‐Si NSs (Figure 4e), and the corresponding videos were also recorded (Videos S1 and S2, Supporting Information). In vitro biological distribution showed that 5‐Fu‐Si NSs can be reduced and cleared after 24 h in most tissues (Figure S5, Supporting Information). After confirming the accumulating effect of 5‐Fu‐Si NSs in tumors in vivo, we then evaluated the thermo‐chemotherapy effects of 5‐Fu‐Si NSs in vivo. Mice bearing *U87‐MG* tumors were exposed to an 808 nm laser (1.5 W cm^−2^) 5 h after intravenous injection. Temperature changes in the tumor region were monitored by infrared thermography. For the 5‐Fu‐Si NSs group, the temperature in the tumor area rapidly increased by 12°C within 5 min, showing an excellent PTT effect, while the PBS control group showed almost no warming effect. Conversely, there was a slight decrease in temperature due to physiological reasons under anesthesia (Figure 4f,g). Overall, these results suggested that 5‐Fu‐Si NSs is a promising biocompatible thermo‐chemotherapy reagent for tumor diagnosis and therapy.

### 2.7 In Vivo Tracking and Evaluation of the Microvascular Changes by OCTA/LS Imaging

**Figure 5:**
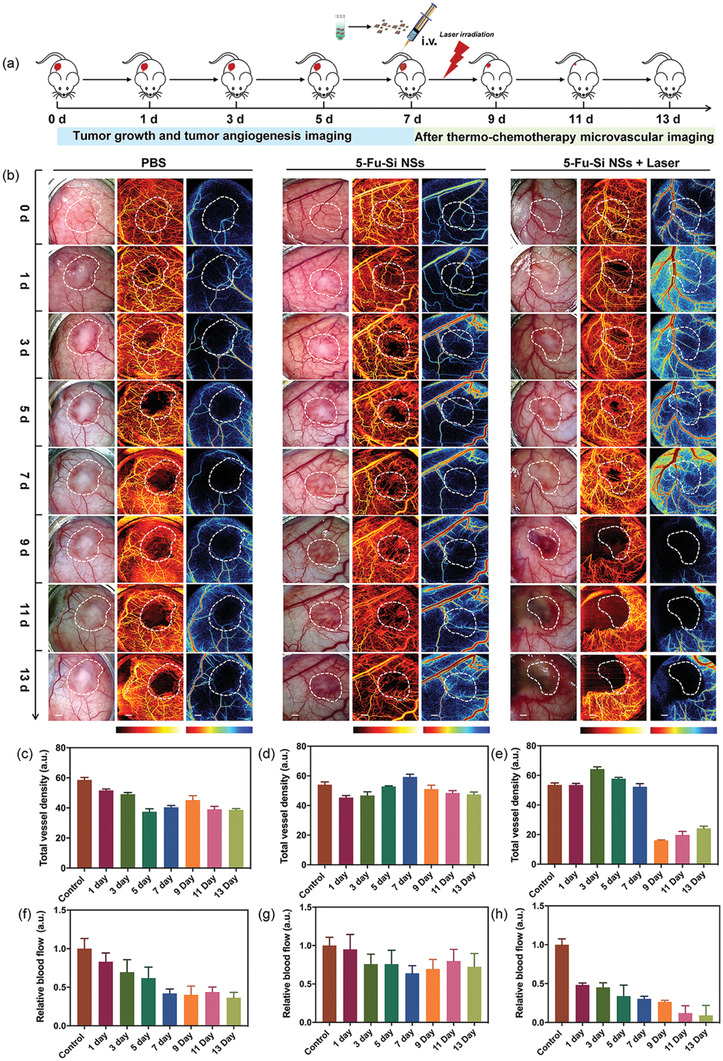
a) Schematic diagram of the model, treatment, and monitoring regime used in mice with 5‐Fu‐Si NSs. b) Representative white light, OCTA, and LS images at different time‐points for each group. The white dotted line represents the tumor area and the ROI area. c) Quantified total vessel density with PBS treatment. d) Quantified total vessel density with Si NSs treatment. e) Quantified total vessel density with 5‐Fu‐Si NSs plus laser irradiation treatment. f) Quantified RBF with PBS treatment. g) Quantified RBF with Si NSs treatment. h) Quantified RBF with 5‐Fu‐Si NSs plus laser irradiation treatment. Scale bar: 2 mm.

Microvascular is the only channel to convey nutrients and remove wastes in tissues. Especially, it plays a significant role in tumor growth and metastasis. Given that, to investigate the tumor angiogenesis and microvascular morphometric changes during the tumor early growth and thermo‐chemotherapy, we performed a back skin window on BALB/c nude mice bearing *U87‐MG* glioma for long‐term OCTA and LS imaging (**Figure** **5**a). Figure 5b displayed the representative white light, OCTA, and LS images (from left to right column), which were collected from one control group (PBS) and two experimental groups (5‐Fu‐Si NSs and 5‐Fu‐Si NSs plus laser) for a total of 13 days. As the tumor grew, the microvessels in the tumor area (marked with dotted white line) gradually dwindled from the center to the edge in the control group before treated with PBS (Figure 5c). And, based on this phenomenon, the RBF in the central area of the tumor was also decreased (Figure 5f). The collapse of blood vessels within the tumor was probably caused by the rapid proliferation of cancer cells in a relatively confined space.^[^ ^42^ ^]^ On the seventh day, PBS solution was i.v. injected into the tumor‐bearing nude mice to serve as a control group. After that, the blood vessels in the ROI of the tumor maintained the characteristics of lower MVD and RBF. On the other side, with the rapid growth of tumor tissue, the tumor cells will secrete plentiful vascular endothelial growth factor to prompt the neovascularization for supplying sufficient oxygen and nutrients that tumor cells need to reproduce.^[^ ^43^, ^44^ ^]^ As shown in LS images of the control group in Figure 5b, with the growth of tumor, the structure of blood supply network around the tumor was changed with neovascularization from preexisting blood vessels, where the blood vessels in LS images were color‐coded to represent RBF from red (higher) to blue (lower). In addition, compared with the early growth stage of tumor (0–3 days), the RBF around the tumor area at the later growth stage (9–13 days) relatively increased to maintain the growth of tumor cells. For the experimental group treated with 5‐Fu‐Si NSs, in the center of solid tumor, we also observed microvascular changes that were similar to the control group, including lower MVD and RBF (Figure 5d,g), before i.v. injection of 5‐Fu‐Si NSs. Subsequently, after i.v. injection of 5‐Fu‐Si NSs (7–13 days), in the ROI of tumor, we observed several neovessels, and the MVD and RBF slightly increased (Figure 5d,g). These results indicated that, as a single chemotherapy method, 5‐Fu‐Si NSs, to some extent, inhibited the rapid growth of tumor cells and relatively alleviated the tissue pressure within the solid tumor, which allowed the recovery of blood vessels inside the tumor. Besides, the microvascular network destroyed by tumor was not restored completely, and the RBF around tumor was relatively higher than that of an early stage of the tumor, which means that the tumor cells were not completely cleared by 5‐Fu‐Si NSs during our experiment. Furthermore, we monitored and evaluated the microvascular changes of the animal tumor model under the treatment of thermo‐chemotherapy strategy (i.v. 5‐Fu‐Si NSs plus laser irradiation). As can be seen in Figure 5b, before the thermo‐chemotherapy treatment, the original vascular structure was destroyed at the center of the solid tumor, leading to the RBF of ROI obvious decrease (Figure 5h). Additionally, the periphery inside the tumor area contained dense neovessels (Figure 5b), which caused a slight increase in MVD (Figure 5e). After thermo‐chemotherapy treatment, the tumor tissues and involved blood vessels were eliminated. What's more, the blood vessels around the tumor area were gradually restored to supply oxygen and nutrients for repairing the damaged tissues (9–13 days).

In sum, our results indicated that OCTA/LS imaging technology was capable of precise tracking and evaluation of the microvascular changes in vivo during tumor growth and after treatments, and can be a powerful tool for providing valuable information on tumor diagnosis and therapeutic effect to doctors.

### 2.8 In Vivo Pharmacokinetics

**Figure 6:**
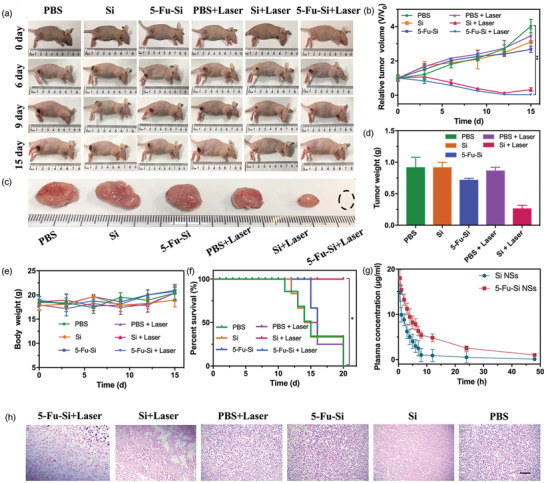
a) Representative photographs of *U87‐MG* tumor‐bearing mice in various treatments. b) Relative tumor volume in different groups (*P < 0.05, **P < 0.01). c) Representative photographs of the tumors collected from different groups of mice at the end. d) Excised tumor weight. e) Body weight of *U87‐MG* tumor‐bearing mice for each group. f) The survival rate during the different formulation treatments. g) In vivo pharmacokinetics profiles of Si and 5‐Fu‐Si NSs in Sprague‐Dawley (SD) rats. h) H&E staining of tumor tissues collected from mice at end point. Scale bar: 100 µm.

Owing to the improved biocompatibility with surface fluorouracil modification, the 5‐Fu‐Si NSs may have a longer circulation lifetime than that of Si NSs. To further verify that, the in vivo pharmacokinetics were explored by i.v. injection of Si NSs and 5‐Fu‐Si NSs into SD rats. As shown in **Figure** **6**g, in SD rats, the plasma concentration of 5‐Fu‐Si NSs attenuated much more slowly over time than that of Si NSs. These results further confirmed that 5‐Fu‐Si NSs have a longer circulating life in blood, and have better drug properties than Si NSs, which will be beneficial to enhance the enrichment and permeability of 5‐Fu‐Si NSs in the tumor sites.

### 2.9 In Vivo Antitumor Activity of Thermo‐Chemotherapy

The above studies confirmed the therapeutic effect of 5‐Fu‐Si NSs in vitro, to further demonstrate that the superior accumulation and penetration of 5‐Fu‐Si NSs could realize enhanced antitumor activity and synergistic thermo‐chemotherapy in vivo, *U87‐MG* tumor‐bearing nude mice were randomly divided into six groups as follows: 1) PBS, 2) PBS plus laser, 3) Si NSs, 4) Si NSs plus laser, 5) 5‐Fu‐Si NSs, and 6) 5‐Fu‐Si NSs plus laser. As shown in Figure 6a–d, 5‐Fu‐Si NSs had inhibitory effect on tumor growth, but the effect was not significant. In sharp contrast, 5‐Fu‐Si NSs plus laser had a higher inhibitory effect on tumor growth. Although the group of Si NSs plus laser had the inhibitory effect, secondary growth occurred after ablation for a period of time due to the lack of chemotherapy drugs to clear the residual tumor tissue. Notably, there was no significant change in the body weight of the tumor‐bearing nude mice with 5‐Fu‐Si NSs during the treatment period (Figure 6e), and no tumor‐bearing nude mice were found to die during continuous follow‐up in this group (Figure 6f).

Next, in order to verify in vivo biosafety of these NTAs, the pathobiology of the above major organs involving the heart, liver, spleen, lung, and kidney was also evaluated by hematoxylin and eosin (H&E) staining. As shown in Figure S6, Supporting Information, the primary organs of tumor‐bearing nude mice treated with 5‐Fu‐Si NSs showed no obvious abnormalities, which clearly indicated that 5‐Fu‐Si NSs were safe in vivo. In addition, H&E staining of tumor sections revealed structural damage in the tumors, and the cancer cell density in the treatment group was significantly reduced. The nuclei were shrunk, and the tumor cells were destroyed (Figure 6h). All of these results suggested that 5‐Fu‐Si NSs can be used as a potential PA imaging agent to achieve effective PA‐guided cancer therapy by combining the advantages of chemotherapy and PTT, as well as to achieve long‐term high‐resolution tumor monitoring under microscopic OCTA and LS imaging strategy. Therefore, 5‐Fu‐Si NSs with biocompatibility are expected to be used in the diagnosis and treatment of tumors as photon induction therapy drugs.

## 3 Conclusion

In summary, a novel multimodal optical contrast nanoplatform developed by weakly interacting force enables on‐demand drug release and synergistic thermo‐chemotherapy in vivo. Benefit from the 2D physical properties of the material, the released drugs showed excellent in vitro antitumor activity. More interestingly, systematic and in vivo studies have shown that due to enhanced tumor enrichment and effective drug delivery, 5‐Fu‐Si NSs not only has the effect of photoacoustic imaging, but also can achieve long‐term microscopic monitoring of tumors under the guidance of photoacoustic, and also can realize good tumor ablation under light induction. Hence, 5‐Fu‐Si NSs is a promising NIR photon agent for clinical translation with excellent therapeutic efficacy and biosafety.

## 4 Experimental Section

### Materials for the Synthesis of Nanoparticles

Li_3_Si_4_ alloy particles were purchased from China Energy Lithium Co., Ltd., and *N*‐methyl pyrrolidone (NMP) were purchased from Sinopharm Chemical Reagent Co., Ltd. Dimethyl sulfoxide (DMSO) and Methylthiazolyldiphenyl‐tetrazolium bromide (MTT) were purchased from Sigma‐Aldrich Co., Ltd. Calcein‐AM and propi‐dium iodide (PI) were purchased from Thermo Fisher Scientific (Waltham, MA) Ltd. Deionized (DI) water (18 MΩ‐cm) was collected from a Milli‐Q Gradient purification system. Unless otherwise stated, all chemical reagents were analytical grade, commercially available, and exploited as received without further purification. Phosphate buffer saline (PBS), Foetal Bovine Serum (FBS), and FA‐scarce RPMI 1640 (RPMI 1640 medium without FA) were obtained from Hyclone. DI water was used throughout.

### Synthesis and Characterization of 5‐Fu‐Si NSs NTAs

2D silicon nanosheets were synthesized via a simple approach coupling chemical delithiation and cryogenic exfoliation. First, 1 g of Li_3_Si_4_ alloy particles were slowly added into 250 mL of anhydrous alcohol in ambient conditions. A lot of H_2_ and heat were generated during the reaction process upon 2 h continuous magnetic stirring. After that, 50 mL of acetic acid was added into the mixture for neutralization, followed by centrifugation (3000 rpm, 10 min) to obtain Si powders composed of stacked silicon sheets. Then, the obtained Si powders (100 mg) were soaked into liquid nitrogen for 1 h. The cryo‐pretreatment powders were immediately transferred into an agate mortar and ground for 40 min for granular refinement. Then, the Si powders and PVP (200 mg) were added into 20 mL of NMP, followed by alternate bath (150 W) and probe (600 W, 50%, period of 3 s with interval of 3 s) sonication for both 2 h under ice bath, respectively. After centrifugation (3000 r min^−1^, 15 min) for isolating the unexfoliated Si powders, PVP‐capped silicon nanosheets were separated by further centrifugation (8000 r min^−1^, 15 min) of the supernatants and washed with DI water 3 times. The morphology and size of 5‐Fu‐Si NSs were recorded using a TEM carried out on a JEOL 1200EX transmission electron microscope at 100 kV. Optical absorption spectra were obtained by a UV–vis spectrometer (Agilent Cary60, USA). Element mapping and element distribution were performed on high‐resolution TEM (HRTEM, FEI, Talos 200s) conducted at 200 kV. The diameter and zeta potential were determined via DLS (Zetasizer 2000, Malvern, UK) and electrophoretic light scattering (ELS) using a Malvern Zetasizer 2000 (Malvern Instruments, Malvern, UK), and the whole of the data were exhibited by mean ± standard deviation (SD). The compositions of the Si‐NSs and 5‐Fu‐Si NSs were confirmed using the Flourier transform infrared spectra (FT‐IR, Bruker IFS‐55 infrared spectrometer). The in vitro physical stability of 5‐Fu‐Si NSs was measured within PBS (PH 7.4), DMEM, and RPMI1640 medium involving FBS for 7 days. The physical stability of 5‐Fu‐Si NSs in different physiological media for predesigned incubation time intervals (≈24 h) was evaluated by determining the diameter, zeta potential.

### Drug Loading and Releasing

Besides, for 5‐Fu loading experiment, 5‐Fu (5 mg) was dispersed in H_2_O (20 mL) for 24 h to obtain 5‐Fu‐encapsulated Si NSs (5‐Fu‐Si NSs). 5‐Fu LC was calculated using a standard calibration curve by Equation (1):

(1) $$LC_{5-\mathrm{FU}}=w_{5-\mathrm{FU}}/w_{\mathrm{Si}}$$

where *W*_5‐Fu_ is the loading weight of 5‐Fu, and *W*_Si_ is the actual mass of the Si NSs. For photo‐stimulated drug release, the release percentages of 5‐Fu were calculated by the absorption spectra of the PBS using a standard curve of absorbance at different concentrations of 5‐Fu. The release percentages (RP, w/w %) were evaluated by Equation (2):

(2) $$\mathrm{RP}\;=w_{\mathrm{released}\;5-\mathrm{FU}}/w_{5-\mathrm{FU}}$$

where *W*_5‐Fu_ is the loading weight of 5‐Fu, and *W*_released 5‐Fu_ is the mass of the released 5‐Fu in the supernatant.

### In Vitro Cell Culture and Cytotoxicity

Human malignant glioma *U87‐MG* cells were obtained from the Institute of Biochemistry and Cell Biology (Shanghai, China), and cultured in DMEM/high glucose medium containing 10% fetal bovine serum (FBS) and 1% antibiotic solution at 37 °C in a humidified atmosphere comprising 5% CO_2_. The cells were first inoculated in 96‐well plates with 1 × 10^4^ cells per well for 12 h, and then washed off after 4 h culture. Add fresh medium containing silicon and incubate for 2 h. The concentrations were 0, 10, 25, 50, 100, 200, 500 µg mL^−1^. After 1 W cm^−2^ of 808 nm laser irradiation for 10 min, the culture base was removed, and 10 mL MTT (0.5 g L^−1^) was added. After incubation for 4 h, the culture medium was removed and added. The absorbance of 490 nm per well was determined by 200 mL DMSO. The cell viability was measured by the standard MTT assay.

### In Vitro Living/Dead Cells Staining Assay

5‐Fu‐Si NSs‐triggered tumor cell death was further investigated by calcein‐AM/PI staining. *U87‐MG* cells were seeded in a 96‐well plate (8000 per well) for 24 h and then incubated with Si NSs and 5‐Fu‐Si NSs solutions (up to 200 µg mL^−1^) for an additional 24 h. Photothermal and chemo‐photothermal groups were irradiated with an 808 nm laser for 10 min (1.0 W cm^−2^). The other groups were incubated under the same conditions without irradiation. Cell viability was evaluated using MTT assays. Living and dead cells were stained with calcein‐AM and PI, respectively. Cell culture medium involving Si NSs and 5‐Fu‐Si NSs were used for culturing *U87‐MG* cells (1.0 × 10^5^ cells per well) for 4 h. Subsequently, living and dead cells were stained by a live/dead staining kit under the manufacturer's instructions and then observed by a fluorescence microscope (Nikon Eclipse Ti‐U, Nikon Inc.).

### In Vitro Assessment of Photothermal Performance

To assess the photothermal effect, 5‐Fu‐Si NSs dispersions (0, 25, 50, 100, 250, 500 µg mL^−1^) were irradiated by 808 nm laser (1.5 W cm^−2^) for 10 min. DI water was used as a control. The temperature of dispersions during irradiation was recorded by a digital FLIR A5 camera with BM IR software (FLIR Systems, Inc., USA) every 30 s. And then under different irradiation power (0.5, 1.0, 1.5, 2 W cm^−2^), the heating effect of the 5‐Fu‐Si NSs was recorded. In addition, to evaluate the photothermal stability, 5‐Fu‐Si NSs dispersions were continuously irradiated by an NIR laser (808 nm, 1.5 W cm^−2^) for 10 min. After cooling for 10 min, continue to irradiate 5 times. The temperature of 5‐Fu‐Si NSs dispersions was recorded during five repeated laser turning on/off cycles. To further measure photothermal conversion efficiency (45.8%), 0.2 mg mL^−1^ of 5‐Fu‐Si NSs aqueous solution was introduced into a quartz cuvette and irradiated with 808 nm laser, and then cooled to room temperature. After five cycles of heating/cooling, there was almost no effect on the photothermal conversion behavior. All the results were recorded for calculating the photothermal conversion efficiency.

### Back Skin Window of *U87‐MG* Tumor Mode

All mice were executed according to the Institutional Animal Care and Use Committee approved procedure at Xiamen University. BALB/c nude mice (5 weeks, male, 22–25 g) were obtained from Xiamen University Laboratory Animal Center. The back skin window was installed in the imaging area, as far as possible to ensure that the imaging area right in the middle. In order to prepare a dorsal dermal chamber consisting of two symmetrical frames, the weight of the mouse was between 22 and 25 grams in mice. The backs of anesthetized mice were carefully shaved and chemically removed to avoid minor damage to the skin. The nude mouse was then placed in a prone position with its back exposed to a medical disinfectant spray. The mouse skin was stretched and examined under clear light to align the main blood supply with the blood vessels. The skin in the middle was sewn together with two 5‐0 threads. The first frame in the chamber was sewn by 5 fixed stitches on its upper back skin frill and carefully prepared by two openings on the bottom of the skin frill close to the body, which was threaded through its connecting screws backward to the front. After marking the circular area of the viewing window, the animals were placed laterally under a stereoscopic microscope. A layer of skin and subcutaneous tissue lipid muscle, as well as two layers of muscle retractors, were completely removed from the area using microsurgical instruments. Therefore, it was of great significance to remove the area outside the observation window (≈11 mm in diameter) and avoid tissue extrusion after the second cavity block was positioned. In addition, special care should be taken when removing the second retractor muscle, which was in direct contact with the next muscle for subsequent microanalysis and should not be damaged. The connecting screw was then placed 400–500 µm from the second frame cavity and the stainless‐steel nut as a locator to prevent compression of the arteriole supplying the vena cava. During the whole implantation process, the surgical site was kept moist with normal saline at 37 °C to avoid tissue drying. Finally, a glass sealed cavity system was placed in the observation window of the second frame. The observation window of the second frame was equipped with a clasp to provide a direct micro‐entry for the microcirculation of the cavity. As can be seen from normal daily feeding, cleaning, and sleeping habits, the animals were well tolerated, no different from animals without models. In the following time, the authors paid close monitoring to the tumor angiogenesis microcirculation in the imaging area.

### In Vitro and In Vivo PA Imaging

To ascertain the PA effect of 5‐Fu‐Si NSs, the 5‐Fu‐Si NSs aqueous solutions with different concentrations of 5, 10, 25, 50, and 100 µg mL^−1^ were added into the agarose in various Eppendorf tubes and fixed into the water tank for PA imaging. And then, the changes of PA signals were acquired at laser wavelength 808 nm by hybrid PA/Ultrasonic imaging system (VevoLAZR‐X, VisualSonics). The energy of a single laser pulse is approximately 36 mJ with 20 Hz pulse rate. Female BALB/c nude mice were purchased from Shanghai Slac Laboratory Animal Co., Ltd, and raised on Xiamen University Laboratory Animal Center before the experiment. Animal experiments were carried out according to the protocols approved by the Animal Care and Use Committee of Xiamen University. In vivo PA imaging was conducted on the same imaging system. Typically, when the tumors grew to ≈100 mm^3^, the tumor‐bearing nude mice were injected intravenously (i.v.) with 200 µl of Si NSs (50 mg kg^−1^) and 5‐Fu‐Si NSs (50 mg kg^−1^) via the i.v. (*N* = 3 per group). After injection, the mice were anesthetized with 2% isoflurane with a gas vaporizer (RWD Company, Shenzhen, China) and scanned with the hybrid system. The real‐time PA signals and SaO_2_ images within the tumor tissues were gained at 0, 2, 5, 8, 12, and 24 h post i.v. injection. The PA signals were recorded in the range of the laser wavelength 680–900 nm. After i.v. injection for 24 h, the tumor‐bearing nude mice were immediately sacrificed using the euthanasia method, and subsequently, the main organs and tumor area tissues were separated. The PA images collected from ex vivo organs and tumor tissue were further pharmacokinetics analyzed. All the data obtained were analyzed using VevoLAB‐3.1 software, and the changes in PA signals, SaO_2_, and HbT were quantified for tumor property assessment.

### In Vivo OCTA Imaging

Subsequently, to obtain a better understanding of how different treatment approaches (PBS, 5‐Fu‐Si NSs, and 5‐Fu‐Si NSs plus laser) to the effects on tumor therapy, the entire microvascular morphological changes during tumor growth and thermo‐chemotherapy were investigated at the micro level using a home‐built swept‐source OCT angiography system. The system light source used a high‐speed swept laser, with the center wavelength of l060 nm and repetition rate of 200 kHz with the full width at half maximum of 100 nm. Briefly, this system can provide a wide field of view (FOV) of about 10 mm × 10 mm with 400 A‐lines in each B‐scan (fast axis X direction). The C‐scan (slow axis Y direction) contained 400 B‐scan with four repeated scans at the same B‐scan area. More details of the OCTA system can be found in Figure S1, Supporting Information. The OCTA experiments were divided into three groups: PBS, 5‐Fu‐Si NSs, and 5‐Fu‐Si NSs plus laser (*N* = 3 per group). For the tumor therapy group, 5‐Fu‐Si NSs and 5‐Fu‐Si NSs plus laser were given to the mice by tail intravenous injection one time starting from day 7 after the tumor size about ≈100 mm^3^, while the untreated tumor group received the same amount of PBS. Before OCTA imaging, a photograph of a tumor area was obtained by an optical microscope. And then, the mice were anesthetized with the same procedure as the above‐mentioned PA imaging. During the OCTA imaging, the mouse was fixed on a high precision translation stage to acquire the optimal imaging location and reduce the motion artifacts. A heating pad was utilized to maintain the body temperature of the mouse during imaging. Subsequently, the tumor microvascular images were captured at 0, 1, 3, 5, 7, 9, 11, and 13 days, respectively. The changes in tumor vascular morphology and MVD were processed using MATLAB (MathWorks Inc., Natick, Massachusetts) by a high‐performance computer workstation. The en face maximum intensity projection images were used to represent the 3D image with dedicated image processing software (Optoprobe science LTD, VCCcular) for complex data further analysis.

### In Vivo LS Imaging

Finally, to further evaluate the vascular response to different treatment strategies (PBS, 5‐Fu‐Si NSs, and 5‐Fu‐Si NSs plus laser), microcirculation changes in the dorsal skinfold tumor model in real‐time were monitored, before and post‐thermo‐chemotherapy by a home‐built LS system. The system laser light utilized a wavelength of 780 nm. All images were collected by a CMOS sensor with the resolution of 1472 × 1104 pixel, FOV range of about 12 × 15 cm, and imaging frame rate of 20 fps. After anesthesia (as detailed above), the distance between the CMOS and dorsal skinfold window was adjusted to the optimal position for LS imaging. All the mice were fixed on the 3D translation stage. In vivo LS imaging was performed during tumor early growth (0, 1, 3, 5 days) as well as after therapy (7, 9, 11, 13 days) in different groups (*N* = 3 per group). Bright‐field imaging and LS imaging were performed according to the same protocol as described in OCTA and PA imaging section. Further image processing was completed using MATLAB (R2016b). The changes in microvascular morphology and RBF were computed to assess the tumor vascular response before and under treatment.

### Imaging Processing and Quantitative Analysis

**Figure 7:**
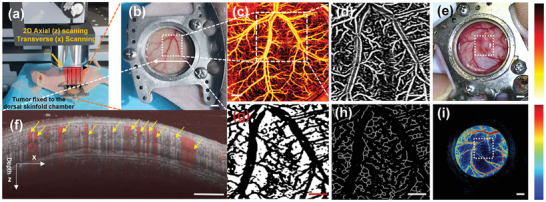
The flow diagram of the microvascular OCTA/LSI imaging and quantification. a) the back skinfold window chamber of *U87‐MG* tumor‐bearing nude mice and OCTA scanning. b) the zoom‐in skin window chamber image in the *U87‐MG* tumor‐bearing nude mice. c) The in vivo corresponding enface microvascular image indicated in (b). d) The enlarged enface microvascular grayscale image of the ROI region indicated by the white dashed box in (b) with FrangiFilter2D filter. e) the representative image of tumor and ROI region indicated by the white dashed box. f) the representative combined tissue cross section structural image (grey color) and blood flow image (yellow arrows). The imaging depth is 2 mm. g) feature map after the thresholding processing. h) extract the blood vessel skeletons image by the OTSU algorithm. i) the representative LSI image and ROI region selected by the white dashed box for RBF quantification. Scale bar: 2 mm in (b,c), 1 mm in (e,i); 1.5 mm in (f); 0.8 mm (d,g,h).

First, to quantify the changes of tumor microvascular parameters with different treatment methods in the back skin window of *U87‐MG* tumor model (**Figure** **7**a), a custom analysis program was written to analyze tumor vascular dynamics from the original OCTA image (10 × 10 mm) with a region‐of‐interest (ROI) of 5 × 5 mm area on the tumor (Figure 7b). More details of the feature map calculation can be found in.^[^ ^45^ ^]^ And then, all the raw OCTA blood vessel images were executed grayscale conversion. Afterward, median filtering and contrast enhancement were used to improve the signal‐to‐noise ratio (SNR) of grayscale OCTA images. Finally, all the grayscale images were first converted into the index images through MATLAB (R2016b) (Figure 7c).

In order to obtain high‐quality tumor region blood vessel segmentation images, FrangiFilter2D filter was used to optimize the grayscale image of the ROI region (Figure 7d). Figure 7f showed the representative combined tissue cross‐section structural image (grey color) and blood flow image (yellow arrows). In the structural image as shown in Figure 7f, multiple tissue layers were clearly identified. It also gave a better explanation for tumor microvascular imaging with visualization anatomical information (Figure 7f). Next, the filter function Frangi formed based on the Hessian matrix was an edge detection enhanced filtering algorithm that can fully realize the vascular image enhancement and obtain effective blood vessel information.^[^ ^46^, ^47^ ^]^ In the study, the FrangiFilter2D parameter factor and fluctuation gradient step size were set to 0.5 and 5 to gain the optimal images. To accurately capture the subtle signals, the ROI region images extracted from the grayscale images were filtered by the custom Gaussian filter (3 × 3), which was created by the following steps:

(3) $$h_{g}\left(n_{1},n_{2}\right)\;=e^{\frac{-\left(n_{1}^{2}+n_{2}^{2}\right)}{2\sigma^{2}}}\;$$

(4) $$h\left(n_{1},n_{2}\right)\;=\frac{h_{g}\left(n_{1},n_{2}\right)}{\sum_{n_{1}}\sum_{n_{2}}h_{g}}\;$$

where *σ* was the standard deviation of the custom Gaussian filter. Here *σ* was set to 1. Finally, the custom Gaussian filter (3 × 3) was determined to provide appropriate smoothing for OCTA data sets. Figure 7e,i showed the selected representative photograph containing a tumor and corresponding LS, which cover the tumor area for quantifying the changes of total RBF. Besides, the locally adaptive thresholding method was chosen to delineate blood vessels and capture blood vessel details in the presence of some background fluctuations (Figure 7g). However, the further extracted blood vessel skeletons and isolated pixels were removed by the Otsu method (OTSU). Finally, the side length of the unit pixel in the 5 × 5 mm ROI area can be calculated, and the eight‐connected area was used as the blood vessel length calculation standard (Figure 7h). Therefore, the total length of the connected blood vessels in the ROI area can be obtained. The total MVD was defined as the total length of the vessel per unit volume, as given as described in.^[^ ^48^ ^]^

### In Vivo Anti‐Tumor Thermo‐Chemotherapy

To evaluate the performance of synergic thermo‐chemotherapy strategy in vivo, a simplified‐version experiment of thermo‐chemotherapy was conducted as a proof of concept. *U87‐MG* tumor‐bearing nude mice established by subcutaneous injection of *U87‐MG* cells (≈1 × 106 in 0.1 mL PBS solution) into the back of each mouse were used as model animals. When the tumor volume reached about 100 mm^3^, the tumor‐bearing nude mice were stochastically divided into six groups (*n* = 5 per group): PBS, PBS plus laser, Si, Si plus laser, 5‐Fu‐Si, and 5‐Fu‐Si plus laser. For the PTT groups, the tumors were irradiated under 808 nm laser (1.5 W cm^−2^, 10 min) at 5 h after tail vein injection. The thermal images were monitored with a FLIR A5 camera and BM IR software. Body weights and tumor sizes were recorded every two days for half a month after the corresponding treatments. The tumor volume was calculated as *V* = (tumor length × tumor width^2^)/2. The relative tumor volume was calculated as *V*/*V*_0_ (*V*_0_: the initial tumor volume) at the first day of the therapy period. The body weight of every mouse was recorded every two days for three weeks via using an electronic balance. Mice in each group were observed until death for measurement of survival percentage and complete curve rate. After 20 days of therapy, all mice were sacrificed via the euthanasia method. The subcutaneous tumors and main organs including heart, liver, spleen, lung, and kidney were excised, weighed, and cleaned with 0.9% NaCl thrice, which were subjected to standard H&E staining for histological analysis.

### Statistical Analysis

Quantitative results were expressed as mean ± SD value. Statistical analyses were performed using SPSS 17.0 software. The difference between two groups was analyzed by the paired Student's *t*‐test method. The differences among multiple groups were analyzed by ANOVA. *P* < 0.05 was considered to be statistically significant with noting via * (** and *** represent *P* < 0.01 and *P* < 0.001, respectively).

## Conflict of Interest

The authors declare no conflict of interest.

## Supporting information

Supporting Information

Supplemental Video 1

Supplemental Video 2

## Data Availability

Research data are not shared.
