# Supplementary material for: Intravital Whole‐Process Monitoring Thermo‐Chemotherapy Via 2D Silicon Nanoplatform: A Macro Guidance and Long‐Term Microscopic Precise Imaging Strategy
Source: Adv Sci (Weinh). 2021 Jun 24;8(16):2101242. doi: 10.1002/advs.202101242 (PMC8373095; doi:10.1002/advs.202101242)
Supplement: Supplementary file 1 — Supporting Information [file ADVS-8-2101242-s003.pdf]

© 2021 Wiley-VCH GmbH

ADVANCED SCIENCE
Open Access

Supporting Information

for *Adv. Sci.,* DOI: 10.1002/advs.202101242

Intravital Whole-process Monitoring Thermo-Chemotherapy via 2D Silicon Nanoplatform: A Macro Guidance and Long-term Microscopic Precise Imaging Strategy

Doudou Huang, Guangxing Wang, Jingsong Mao, Chunlei Liu, Zhongxiong Fan, Yunrui Zhang, Bei Zhang, Yang Zhao, Cuixia Dai, Yaqin He, Heng Ma, Gang Liu, Xiaoyuan Chen, and Qingliang Zhao**

Supporting Information

Intravital Whole-process Monitoring Thermo-Chemotherapy via 2D Silicon Nanoplatform: A Macro Guidance and Long-term Microscopic Precise Imaging Strategy

Doudou Huang, Guangxing Wang, Jingsong Mao, Chunlei Liu, Zhongxiong Fan, Yunrui Zhang, Bei Zhang, Yang Zhao, Cuixia Dai, Yaqin He, Heng Ma, Gang Liu, Xiaoyuan Chen, Qingliang Zhao**

D. Huang, Dr. G. Wang, Y. Zhang, B. Zhang, Prof. G. Liu
State Key Laboratory of Molecular Vaccinology and Molecular Diagnostics, Center for Molecular Imaging and Translational Medicine, School of Public Health, Xiamen University, Xiamen 361102, China

Prof. J. Mao
Department of Radiology, Xiang'an Hospital of Xiamen University, Xiamen 361102, China

Dr. C. Liu
Laboratory of Translational Medicine, Medical Innovation Research Division of Chinese PLA General Hospital, Beijing, 100853, China

Dr. Z. Fan
Department of Biomaterials, College of Materials, Research Center of Biomedical Engineering of Xiamen & Key Laboratory of Biomedical Engineering of Fujian Province & Fujian Provincial Key Laboratory for Soft Functional, Xiamen University, Xiamen 361102, China

Prof. Y. Zhao
Department of Mechanical and Electrical Engineering, Xiamen University, Xiamen 361102, China

Prof. C. Dai
College of Physics, Shanghai Institute of Technology, Shanghai, 201418, China

R.A. Y. He
Department of Colorectal Surgery, General Hospital of Ningxia Medical University, Yinchuan, 750004, China

Prof. H. Ma
Department of Physiology and Pathophysiology, School of Basic Medical Sciences, Fourth Military Medical University, Xi'an 710032, China
E-mail: hengma@fmmu.edu.cn

Prof. X. Chen
Yong Loo Lin School of Medicine and Faculty of Engineering, National University of Singapore, Singapore, 117597, Singapore

Prof. Q. Zhao
State Key Laboratory of Molecular Vaccinology and Molecular Diagnostics, Center for Molecular Imaging and Translational Medicine, School of Public Health, Xiamen University, Xiamen 361102, China
Shenzhen Research Institute of Xiamen University, Shenzhen 518063, China.
E-mail: zhaoql@xmu.edu.cn

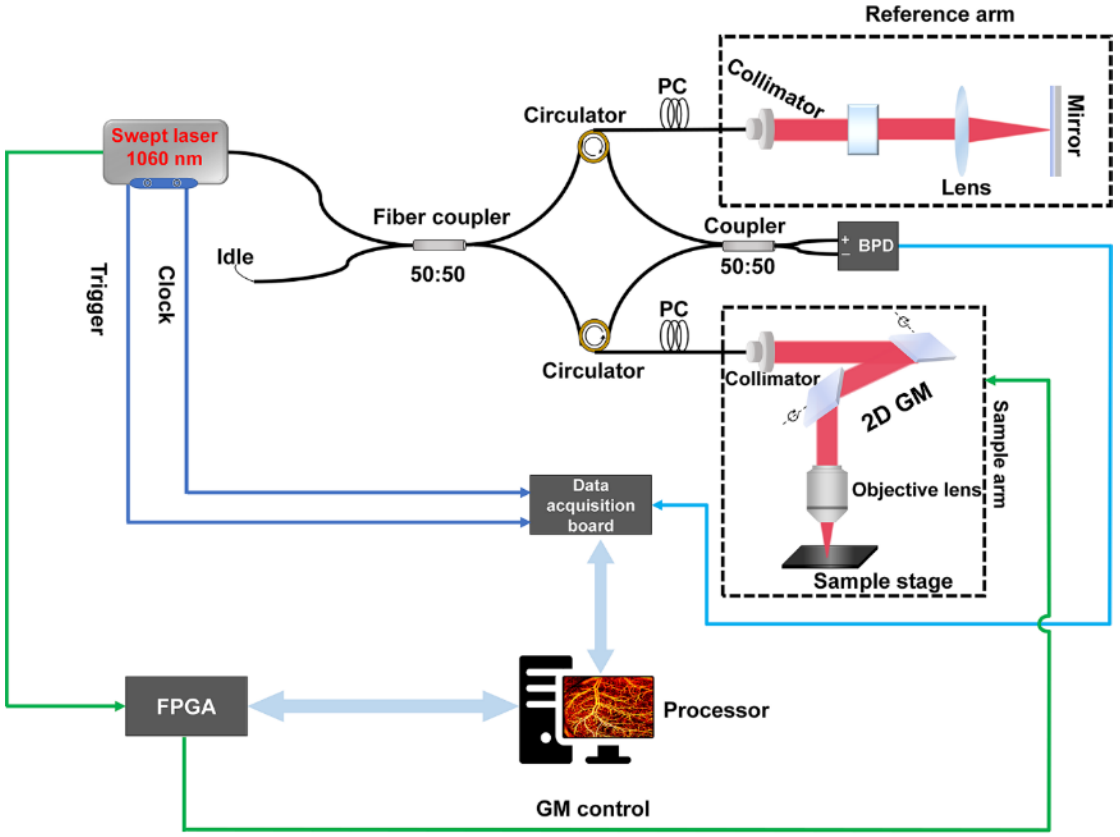

Figure. S1. Schematic diagram of the high resolution OCTA imaging system.

A home-built SS-OCT system was utilized to monitor the changes of microvascular of tumor in vivo. This system employed a high speed swept source light source (Axsun, Inc. USA) at 1060 nm central wavelength, which operated at 200 kHz swept rate with 100 nm tuning spectral range bandwidth. The light was split into a 2× 2 single mode optical fiber via 50:50 coupler, and then the two optical circulators were used to transfer the light into sample arm and reference arm. The return backscattering signal from both arm interferes was detected using a balanced photodetector with 1.6 GHz bandwidth (PDB480C-AC, Thorlabs, Inc.). The interference signal from the detector, the synchronization A-line sweep triggers, and K-clock of the light source were detected and recorded using a high-speed digitizer (12-bit up to 1.8

GS/s, AlazarTech, ATS9360). A high speed of 2D scanning galvanometric mirrors (Cambridge Technology, 6215H) were employed and synchronized with the swept source and controlled by a 12-bit high-speed analog output board (National Instruments, PCI-6713). A scanning object lens (Thorlabs, LSM03BB) with 36 mm long focal length was employed in the sample arm, which provided ~29 μm lateral resolution, and ~5 μm axial resolution. It can provide the maximum FOV about 10×10 mm^2. All the raw 3D datasheet was processed by a host computer (AMD Ryzen 3.6 GHz, 1T RAM, X64 system) through 8-lane PCI Express Gen2 interface. All the control program with a customized built software, which is on LabVIEW™ platform including laser sweeping, galvo-scanning, system synchronization, real-time imaging preview, and data acquisition. Further post-processing of the raw data to yield the images has been performed on MATLAB.

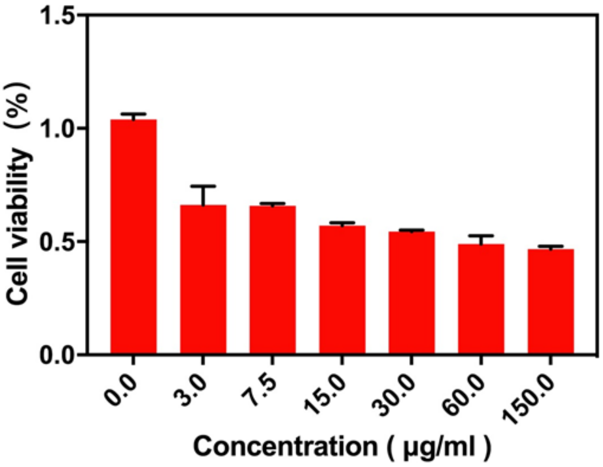

Figure S2. The concentration-dependent cell viability of *U87-MG* cells treated with Si NSs and 5-Fu-Si NSs after 12 h incubation.

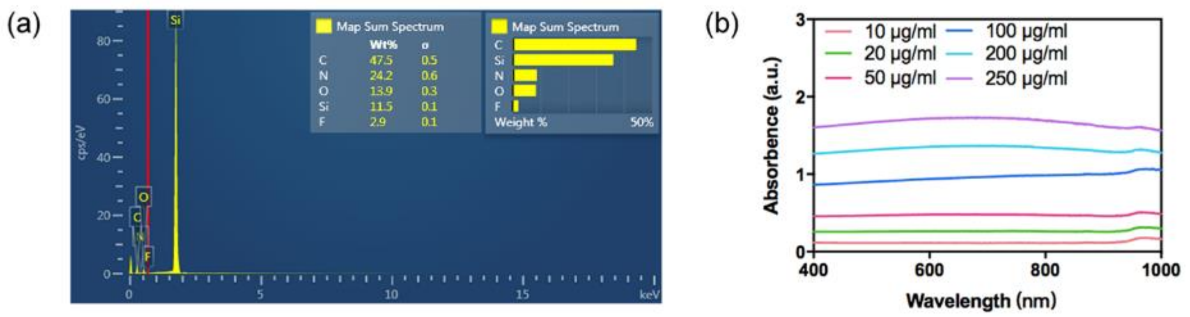

Figure S3. (a) Element distribution of 5-Fu-Si NSs. **(b)** Optical absorption spectra of 5-Fu-Si NSs at different concentration.

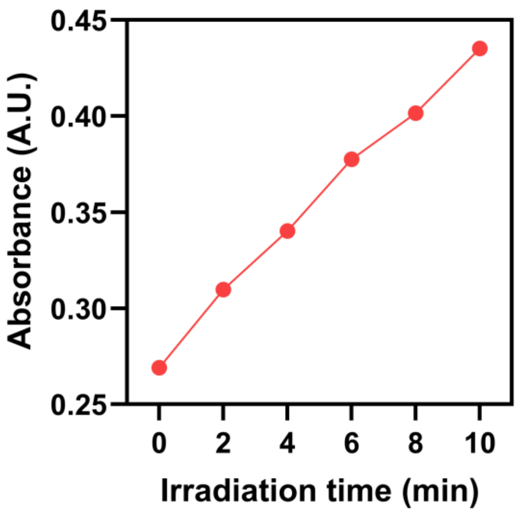

Figure S4. The release of 5-Fu from 5-Fu-Si NSs after the 808 nm laser irradiation (1.0 W·cm^{-2}) with different irradiation time. The absorption peak of 5-Fu is 266nm. Thus, the absorbance of 5-Fu at wavelength of 266nm was selected as an indicator to verify whether laser treatment would accelerate the release of 5-Fu.

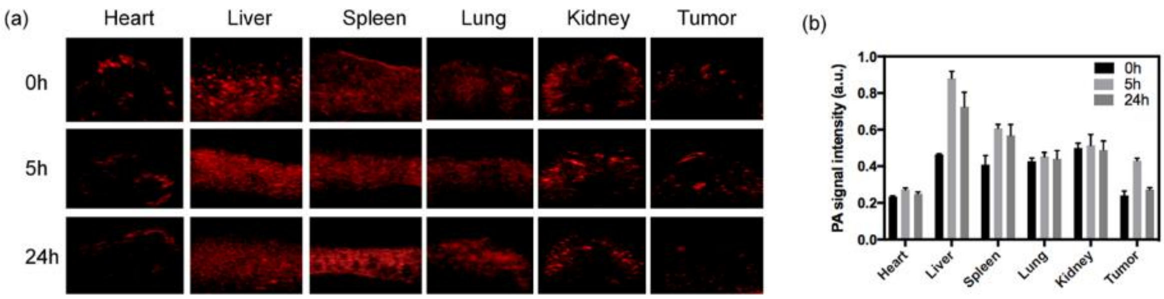

Figure S5. (a) PA images of the organs from the mice of control group (0h) and 5h, 24h after injection of 5-Fu-Si NSs. **(b)** Quantification of PA signal changes of each organs after i.v. injection of 5-Fu-Si NSs.

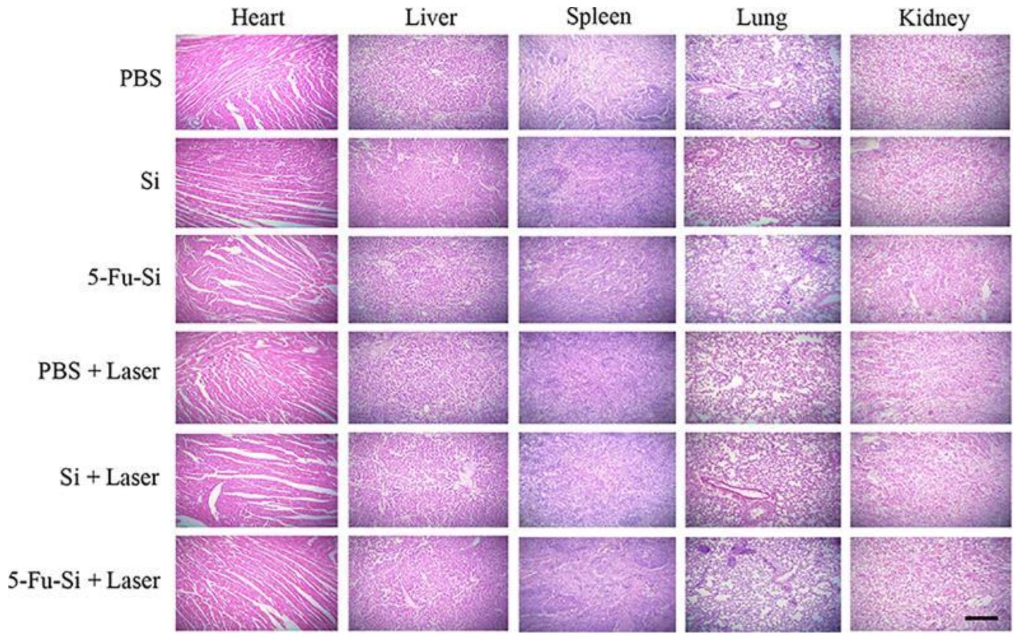

Figure S6. H&E staining images of the major organs collected from each group after receiving different treatments. Scale bar: 100μm.

Supplementary Video S1. Representative schematic diagram of PA 3D scanning video at 808 nm.

Supplementary Video S2. Representative schematic diagram of PA SaO_2 3D scanning video.
